# Supplementary material for: A Novel Ferroptosis Inhibitor UAMC-3203, a Potential Treatment for Corneal Epithelial Wound
Source: Pharmaceutics. 2022 Dec 29;15(1):118. doi: 10.3390/pharmaceutics15010118 (PMC9863691; doi:10.3390/pharmaceutics15010118)
Supplement: Supplementary file 1 [file pharmaceutics-15-00118-s001.zip › pharmaceutics-2057457-supplementary.pdf]

Supplementary Information

Figures

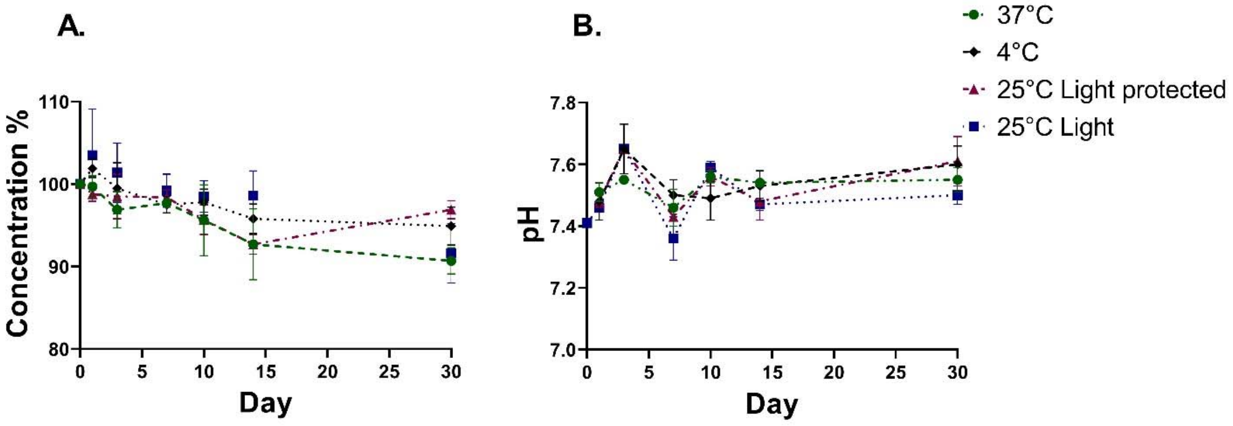

Figure S1. Stability study of UAMC-3203 (100 µM) A) concentration %, B) pH as a function of time. The results are expressed as mean ± standard deviation (SD), n = 3.

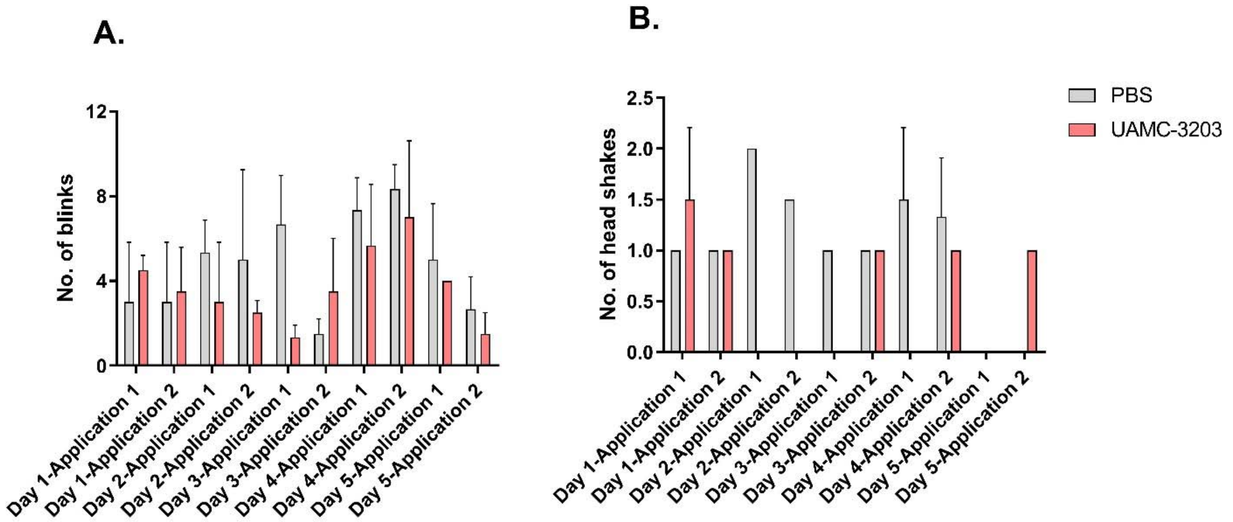

Figure S2. Comparison of number of A) eyeblinks and B) head shakes in control and UAMC-3203 treated eyes during safety studies. The results are expressed as mean ± SD, *n* = 3.

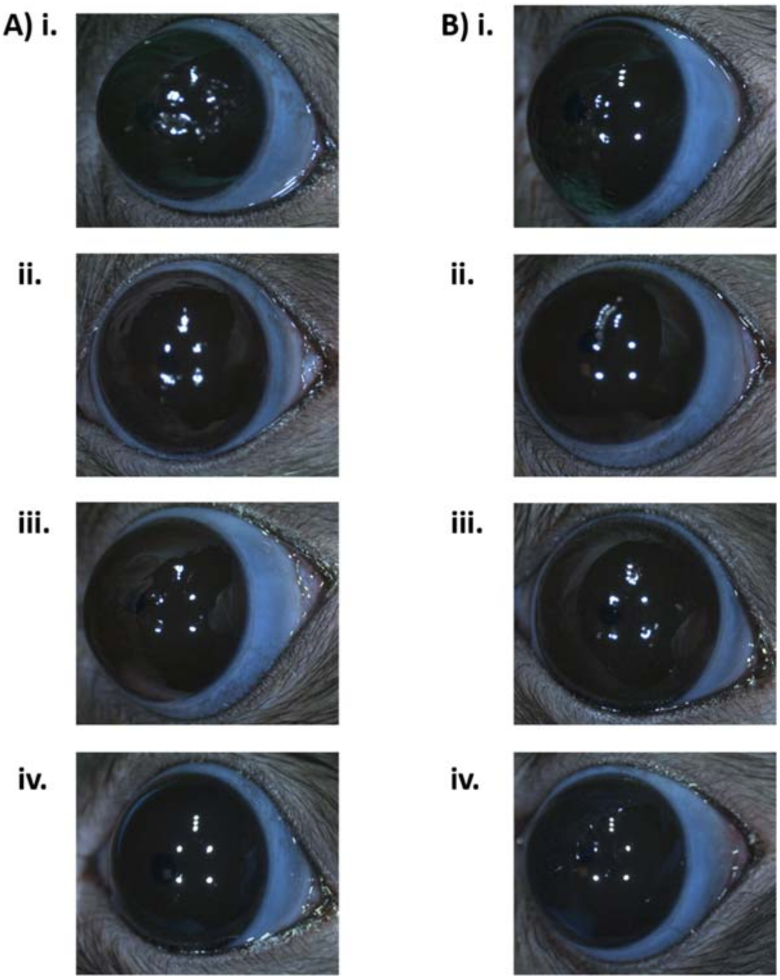

Figure S3. Representative images of cornea and sclera observed during *in vivo* safety study of A) control and B) treated groups at i) baseline, ii) day 2, iii) day 5, and iv) post study (day 8).

Table

Table S1. MS/MS parameters for UAMC-3203 and diclofenac.

Compound	Precursor ion (m/z)	Product ion 1 (m/z)	CE for product ion 1 (V)	Product ion 2 (m/z)	CE for product ion 2 (V)	IS
UAMC-3203	472.3	279.1	30	295	33	Diclofenac
Diclofenac	296	214	40	250	10	-

CE, Collision energy
